# Post‐translational modifications of the cardiac proteome in diabetes and heart failure

**DOI:** 10.1002/prca.201500052

**Published:** 2015-09-14

**Authors:** Adam R. Wende

**Affiliations:** ^1^ Department of Pathology, Division of Molecular and Cellular PathologyUniversity of Alabama at BirminghamBirminghamALUSA

**Keywords:** Cardiomyocyte, Epigenetics, Metabolism, Mitochondria, *O‐*GlcNAcylation

## Abstract

Cardiovascular complications are the leading cause of death in diabetic patients. Decades of research has focused on altered gene expression, altered cellular signaling, and altered metabolism. This work has led to better understanding of disease progression and treatments aimed at reversing or stopping this deadly process. However, one of the pieces needed to complete the puzzle and bridge the gap between altered gene expression and changes in signaling/metabolism is the proteome and its host of modifications. Defining the mechanisms of regulation includes examining protein levels, localization, and activity of the functional component of cellular machinery. Excess or misutilization of nutrients in obesity and diabetes may lead to PTMs contributing to cardiovascular disease progression. PTMs link regulation of metabolic changes in the healthy and diseased heart with regulation of gene expression itself (e.g. epigenetics), protein enzymatic activity (e.g. mitochondrial oxidative capacity), and function (e.g. contractile machinery). Although a number of PTMs are involved in each of these pathways, we will highlight the role of the serine and threonine *O*‐linked addition of β‐*N*‐acetyl‐glucosamine or *O‐*GlcNAcylation. This nexus of nutrient supply, utilization, and storage allows for the modification and translation of mitochondrial function to many other aspects of the cell.

**Abbreviations**
HDAC: histone deacetylase
IFM: interfibrillar mitochondria
I/R: ischemia/reperfusion
OGA: *O*‐GlcNAcase
OGT: *O*‐GlcNAc transferase
OXPHOS: oxidative phosphorylation
SIRT: sirtuin
SSM: subsarcolemmal mitochondria
STZ: streptozotocin
TET: ten‐eleven translocation methylcytosine dioxygenase
T1D: type 1 diabetes
T2D: type 2 diabetes

## 1 Metabolism out of control

**Figure 1:**
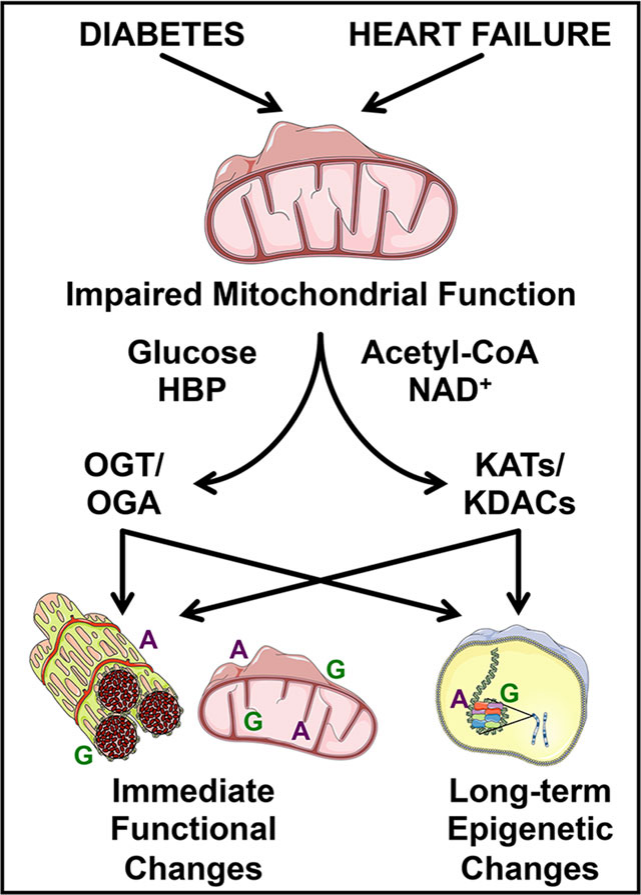
Changes in mitochondrial function and metabolic pathways in diabetes and heart failure alter metabolite driven PTM of proteins. The decreased mitochondrial oxidative capacity results in increased flux through nonmetabolic pathways such as glucose to the hexosamine biosynthetic pathway and altered NAD^+^ and acetyl‐CoA levels. Together these changes alter protein *O*‐GlcNAcylation (G) via *O‐*GlcNAcase (OGA) and *O‐*GlcNAc transferase (OGT) and acetylation (A) via lysine acetyl transferases (KATs) and lysine deacetylases (KDACs). The altered PTMs can acutely regulate function by modifying contractile and oxidative machinery or by modifying chromatin to change gene expression hours, years, and possibly decades later.

Obesity and diabetes occur from a loss of metabolic control indicated by too much nutrient storage and disrupted utilization. The number one cause of mortality in diabetic patients is from cardiovascular complications 1, 2, 3. The development of heart failure itself is also associated with a loss of the ability of the mitochondria to utilize metabolites normally in response to differing levels of nutrient sources 4, 5. This flexibility in substrate utilization is part of normal development as is seen at birth when the mammalian fetal heart switches from a reliance on lactate and glucose to one of fatty acid utilization 6. However, when heart failure develops as a result of hypertension, the heart reverts to its fetal program reducing oxidation of fatty acids and increasing utilization of glucose 7, 8. The opposite can be seen in type 1 diabetes (T1D) and type 2 diabetes (T2D) as a result of altered nutrient uptake, storage, and utilization. Insulin‐resistant states disrupt signaling systemically with numerous changes such as hyperinsulinemia, hyperlipidemia, and hyperglycemia 9. T2D is a chronic condition that results when the body is either resistant to the effect of insulin or progresses to insulin deficiency. It is often associated with obesity however a number of genetic loci have been found in at‐risk populations. T1D is also a chronic condition that can result from an autoimmune response as a result of exposure to certain viruses and genetic risk factors leading to loss of pancreatic β cells and low or absent insulin production. In each of these cases the body is no longer able to properly regulate nutrient utilization leading to increases in circulating metabolites. In this nutrient‐rich environment, the result is a further increase in fatty acid oxidation and despite the hyperglycemia there is a decreased reliance on glucose 10, 11, 12. Drowning in a sea of nutrients, mitochondrial dysfunction ensues and cellular respiration becomes uncoupled from ATP synthesis, leading to generation of excess reactive oxygen species, and cellular damage. Collectively the cellular dysfunction seen in obesity and diabetes from disrupted fatty acid utilization is referred to as lipotoxicity, which has been extensively reviewed 13, 14, 15. In addition to the contribution of altered mitochondrial substrate flux to the diabetic heart, a number of studies have explored the contribution of changes in gene expression for these metabolic switches 16, 17, 18, 19, and this has also been extensively reviewed 8, 20. Although both of the above mechanisms are critical components of the metabolic switch, a missing part of this regulation is the contribution of metabolite‐driven PTMs (e.g. acetylation and *O‐*GlcNAcylation) of nuclear, cytosolic, and mitochondrial proteins 21, 22, 23, 24, 25, 26, 27 (Fig. 1). These metabolically driven PTMs contribute to gene expression, either acutely by modifications of transcription factors 28, 29, 30 or long term by epigenetic mechanisms as a result of PTMs on chromatin proteins (Fig. 1). The purpose of the current review is to highlight how recent advances in our understanding of the PTM regulation of cardiac mitochondrial function and related gene expression may contribute to the development of heart failure in diabetes.

## 2 Diabetes and heart failure

Tissue damage can occur immediately or smolder insidiously for years. A patient might die instantly from an acute myocardial infarction or decline over decades from environmental factors experienced by their parents. Whatever the etiology, this dysfunction can occur at the molecular level and alter the cells ability to repair or regenerate. Conversely it may be a result of an underlying congenital defect or epigenetic programming. In the case of obesity and diabetes, the progression to heart failure is often seen as a result of excess nutrient supply, insufficient nutrient utilization, dysfunctional nutrient storage and oxidation, or a combination of all the above. Defining the molecular mechanisms of these complex chronic diseases has been a challenge and decades of work have gone into defining the transcriptional reprogramming, disrupted cellular signaling, and metabolic physiology. Two new areas that have gained particular interest in defining this problem are metabolomics and proteomics. Advances have occurred in defining the cardiac proteome in unraveling disease 31. However, it is now clear that we are not seeing the whole picture. Understanding disease progression requires the integration of all the above with the metabolite‐driven PTM of the proteome providing the missing piece. PTM regulation of protein function can explain acute changes in contractile, mitochondrial, and transcriptional control as well as explain transgenerational epigenetic regulation of disease susceptibility. *O‐*GlcNAcylation of mitochondrial proteins can be a direct point of metabolic regulation while the addition of GlcNAc to chromatin could explain some inheritance in these diseases. The following subsections will define the metabolic changes specifically seen in the diabetic heart, the failing heart, and how the altered proteome might contribute to this cellular dysfunction and then we will explore the contribution of *O*‐GlcNAcylation to this process.

### 2.1 Metabolic changes in diabetes and heart failure

Sixty years ago researchers found that despite excess glucose in diabetes the heart shows a preferential oxidation of fatty acids 10. The make‐up of the metabolites (e.g. saturated, unsaturated fatty acids), duration of the stress (e.g. high fat meal, obesity, diabetes), and other medical co‐morbidities (e.g. hypertension) all influence the loss of metabolic flexibility. Recent evidence now highlights the possibility that the metabolic switch may precede other pathologies in the development of heart failure 5. Although there is debate on the topic of metabolic remodeling as cause, consequence, or even adaptation 4, 32, 33, 34, we will see below that many of the unbiased proteomic screens identify mitochondrial and more specifically oxidative phosphorylation (OXPHOS) protein regulation involved with disease progression.

### 2.2 Mitochondrial proteome in diabetes and heart failure

Before we can fully understand what PTMs are contributing to the regulation of cardiomyocyte function, we must define the proteome being regulated. A recent attempt to catalog human tissue‐specific proteomes was conducted looking at over 24 000 proteins in 44 tissues 35. Using a combination of immunohistochemistry and RNA‐sequencing the authors discovered tissue‐specific signatures, subgroupings, and common expression patterns. Interestingly, the authors found that 60% of all metabolic enzymes are expressed in all analyzed tissues. With the link between mitochondrial dysfunction and diabetic heart disease we should focus on what changes are occurring within the mitochondrial proteome. The make‐up of the mitochondrial and nuclear‐encoded mitochondrial‐targeted proteins are known to vary between tissues, developmental status, and disease progression. MitoProteome gives one source of information that focuses on the human cardiac mitochondrial proteome 36. MitoCarta on the other hand is a comprehensive comparison of mitochondrial‐targeted proteins from 14 tissues in mice that defines 1098 genes with protein product localization to the mitochondria 37. The MitoCarta has been used to aid in the interpretation of the diabetic liver mitochondria acetylome 38 or translated to the conserved human list in MAGENTA 39. MitoInteractome 40 and MitoMiner 41 are database “warehouses” that bring together a number of current datasets to aid in the interpretation and integration of proteomic analysis. As this is a dynamic process a number of hurdles have stood in the way and a comprehensive review focused on defining the cardiac mitochondrial proteome was published earlier this year 42.

The complexity of these studies does not stop with differences in species, tissue, and organelles in the proteome mentioned above. Added layers found in alterations due to aging and disease progression in diabetes and heart failure further influence the proteins at the mitochondria. Even if we focus on the heart, the subcellular localization and protein trafficking can regulate function. A comparison of mouse tissues from the Akita mouse model of T1D, found the largest differences in the cardiac mitochondrial proteome 43. In another model of T1D, streptozotocin (STZ), these mitochondrial proteome changes were shown to be more pronounced in the interfibrillar mitochondria (IFM) compared to the subsarcolemmal mitochondria (SSM) 44. Interestingly, the same group found that in the db/db mouse model of T2D, SSM had more pronounced differences than IFM 45. Some of these differences in SSM and IFM are also seen in pressure overload induced heart failure and are partially regulated by a bidirectional alteration in protein stability 46. Therefore even different mitochondria within a cardiomyocyte can have different proteomes, calling for some single‐cell analysis in the future. The common theme of altered mitochondrial proteins has also been found in the diet induced prediabetic mouse heart, with increased fatty acid utilization proteins and decreased mitochondrial energetic proteins 47. Alternatively, if insulin signaling is enhanced through transgenic expression of protein kinase b (PKB/AKT), there is a progressive development of heart failure consistent with what is observed in pressure overload induced hypertrophy and associated with a decrease in OXPHOS proteins 48. This decline in OXPHOS and other metabolic proteins was also observed in human patients with dilated cardiomyopathy 49.

Some progress has been made to reverse these changes in the proteome using animal models and has correlated with better function. Changes in the cardiac proteome also occur in response to physiological stimuli, although the targets may differ by mode (e.g. running, swimming) and duration of exercise 50, 51. For example a single bout of exercise was sufficient to produce differential changes in the cardiac proteome of obese ob/ob mice 52. Exercise training of postmyocardial infracted rats preserved cardiac function that was linked to a decrease in voltage‐dependent anion channel 2 (VDAC2) and increases in antioxidant proteins glutathione peroxidase 1 (GPX1) and manganese superoxide dismutase (MnSOD) 53. Transgenic overexpression of a different antioxidant enzyme, the mitochondria phospholipid hydroperoxide glutathione peroxidase 4 (mPHGPx) preserved the cardiac mitochondrial proteome in STZ‐induced diabetes 54. Treating mice with the cell‐permeable mitochondria‐targeted peptide antioxidant, Szeto‐Schiller (SS) tetrapeptide (SS31), prevented 84% of the mitochondrial proteome changes that were otherwise seen in nontreated pressure overload induced heart failure 55. While in humans mechanical unloading using a left ventricular assist device reversed some of the protein changes seen in heart failure 56. Identifying the proteomes and showing that they can be altered with therapeutic utility provides the scaffold on which to overlay the PTMs.

### 2.3 Laying a foundation: Cardiovascular complications in this generation and the next

Most of the protein changes discussed above focused on a response to a stress that was realized during the progression of the disease or immediately following an acute stimulus. However, a number of studies have now implicated epigenetics in the susceptibility to cardiovascular complications of obesity and diabetes. One explanation for the growing prevalence of diabetes in the modern world is the role of epigenetics in regulating gene expression. It is now clear that changes in our environment, diet, and activity level all alter gene expression in an organism and that of its offspring without altering genomic sequence via a process termed epigenetics. Epigenetics includes PTMs to histones and modification of DNA. Although epigenetics can be transgenerational 57, the associated modifications can be dynamically regulated by lifestyle 58. The clinical significance of epigenetic changes in diabetic patients is supported by the findings of the Diabetes Control and Complications Trial/Epidemiology of Diabetes Interventions and Complication study (DCCT/EDIC) 59, 60. Specifically, patients who were continuously on intensive therapy had a significantly lower incidence of cardiovascular complications than patients who were initially on conventional therapy and subsequently switched to intensive therapy 61. These findings support the hypothesis that prior hyperglycemia may lead to long‐lasting molecular changes predisposing patients to the development of complications. This metabolic or glycemic memory is the sustained changes in cellular function as a result of antecedent fluctuations in glucose levels 62, 63. Similar mechanisms also linked epigenetics to T2D as a result of parental over‐ or under‐nutrition 64, 65. Therefore the PTM‐mediated regulation of the cardiac proteome may be a foundation laid earlier in the individual's life or even that of their parents and grandparents. Protein *O‐*GlcNAcylation contributes to the epigenome and is involved in regulation of the metabolic genes and therefore proteomes defined above. Its contributions to the altered mitochondrial function found in the diabetic and failing heart will be included in the discussion below.

## 3 *O*‐GlcNAcylation

### 3.1 Identification and regulation of *O*‐GlcNAcylation

Glycoproteomics is a complex and important area of study for a number of organ systems, including our understanding of cardiac function. This can involve the regulation of extracellular and surface branched glycans 66 and *N*‐linked glycosylation 67, or as described below, a single intracellular *O*‐linked glycosylation. Conserved in eukaryotes from *Caenorhadbditis elegans* to humans, addition of β‐*N*‐acetyl‐glucosamine on serine and threonine residues of proteins via an *O‐*linkage, or *O*‐GlcNAcylation was discovered about 30 years ago 68. One of the largest hurdles in the study of GlcNAc biology has been the site‐specific identification of these modifications. Many of these have been overcome 69, leading to an explosion of research on this PTM that was highlighted by two different journals that dedicated entire thematic review issues 70, 71. It is now clear that *O‐*GlcNAcomics is an important area of study to complete our understanding of numerous diseases 72. Unlike other modifications (e.g. phosphorylation) that have 100s of regulatory proteins, the products of only two genes mediate *O‐*GlcNAc modifications; *O*‐GlcNAc transferase (OGT) that adds a GlcNAc and *O‐*GlcNAcase (OGA, MGEA5, or NCOAT) that removes it. *O*‐GlcNAc signaling is also regulated by flux through the hexosamine biosynthetic pathway that brings together input from glucose, amino acid, fatty acid, and nucleotide metabolism to produce the high‐energy donor and substrate for OGT, UDP‐GlcNAc. This seemingly simple pathway has proved to be critical to both adaptive responses and disease progression in its ability to integrate cellular signaling from multiple metabolic pathways.

The X‐linked *Ogt* gene is essential for embryonic stem cell development 73. Recent evidence has shown that this is true for cardiac‐specific OGT levels as well, with only 12% of constitutive cardiomyocyte‐specific OGT‐null mice surviving to weaning and those that did experiencing severe heart failure 74. Earlier this year, a similar requirement of OGA in development and survival was found in OGA‐null mice 75. As OGA is not X‐linked, heterozygous animals could be studied. This haploinsufficiency led to a modest increase in protein *O‐*GlcNAcylation that was accompanied by a profound alteration in gene expression, with the largest changes seen in genes for immunity and metabolism. When the *Mgea5^−/+^* animals were placed on a high fat diet, there was an exaggerated weight gain observed only in the female mice 75. The cardiac phenotype was not reported on, but it is interesting to speculate that similar changes in metabolic capacity and function would be observed.

Additional evidence linking *O*‐GlcNAcylation to diabetes comes from both rodent and human studies. For example an early linkage study found that T2D age at onset in Mexican Americans mapped to chromosome 10q 76, a region that was subsequently shown to have a single‐nucleotide polymorphism (SNP) in intron 10 of the *MGEA5* gene 77. In the spontaneously diabetic Goto‐Kakizaki rat 78 a transcript variant missing exon 8 that encodes the region for hexosaminidase activity was found 79. This variant has been studied using transgenic overexpression in skeletal muscle 80, and future studies are warranted to see if there is a cardiac phenotype. These observations provide exciting genetic evidence for the link between *O‐*GlcNAcylation and diabetes.

Beyond genetic loci, a number of studies have connected nutrient availability to *O‐*GlcNAcylation levels. For example, sustained high glucose associated with hyperglycemia in diabetes may stimulate hexosamine biosynthesis pathway flux leading to elevated protein *O*‐GlcNAcylation 81. Enhanced glucose delivery to skeletal muscle is sufficient to increase protein *O‐*GlcNAcylation as was seen in response to transgenic overexpression of the glucose transporter GLUT1 associated with insulin resistance and a reciprocal *O‐*GlcNAcylation of the related GLUT4 82. More recent evidence found overexpressing GLUT1 in the heart and subjecting the mice to the transverse aortic constriction model exacerbates pressure overload induced hypertrophy and is associated with increased protein *O‐*GlcNAcylation 83. Specific mechanisms for the ensuing cardiac dysfunction include *O‐*GlcNAcylation of proteins involved in signal transduction, calcium handling, apoptosis, autophagy, proteosomal degradation, contractile machinery, transcription factors, and chromatin modifiers. A few of the more recently identified targets are described here. In mouse, rat, and human cardiomyocytes *O‐*GlcNAcylation of serine 279 of Ca^2+^/calmodulin‐dependent protein kinase II (CaMKII) results in impaired calcium handling and increased cardiac arrhythmia 84. Acute high glucose treatment of primary rat cardiomyocytes or 4 weeks diabetes (STZ) both increased total protein *O‐*GlcNAcylation in cardiac cells, with a requirement of ERK1/2 activation 85. However, in a T2D model of combined high fat diet with a single low dose STZ injection *O‐*GlcNAcylation did not become significant until 2 months 86. In addition to nutrient excess it has also been shown that glucose deprivation can increase protein *O*‐GlcNAcylation often to a higher level 87. This enhancement of the modification at both levels of glucose availability is seen for glycosylation of other proteins. Specifically, a study of 8683 individuals with T2D found that either high or low HbA1c predicted development of heart failure 88. It is tempting to speculate that *O*‐GlcNAcylation may have a similar role in regulating molecular function in times of cellular feast and famine acting as a glucose rheostat and defining its levels at different stages of disease progression requires further investigation.

### 3.2 Mitochondrial protein *O*‐GlcNAcylation

Although once thought to occur primarily on proteins of the cytoplasm and nucleus 89, *O*‐GlcNAcylation has now been found on a number of proteins in the mitochondria 21, 90, 91, 92. In 2002, the first evidence for mitochondrial targeting of the 103 kDa isoform of OGT (mOGT) was presented 93. Using O‐(2‐acetamido‐2‐deoxy‐D‐glucopyranosylidene) amino‐N‐phenylcarbamate (PUGNAc), an inhibitor of OGA, investigators found *O‐*GlcNAcylation of mitochondrial VDAC 94. The increase in VDAC and modification by *O‐*GlcNAcylation is thought to be protective in ischemic/reperfusion (I/R). However, in 2009 it was shown that high glucose is sufficient to increase mitochondrial protein *O*‐GlcNAcylation 21. To map the sites the investigators used a series of studies that included β‐elimination followed by Michael addition with dithiothreitol (BEMAD) and tandem mass spectroscopy. The samples were isolated mitochondria from primary neonatal rat cardiac myocytes treated with either 5.5 mM low glucose or 30 mM high glucose. *O‐*GlcNAcylated proteins were identified in complexes I, II, and III of the OXPHOS pathway. Specifically one GlcNAcylated site is serine 156 of NADH dehydrogenase (ubiquinone) 1α subcomplex, 9 (NDUFA9) of OXPHOS complex 1. Using enzymatic activity assays, this elevated PTM was associated with decreased complex activity that could be rescued when OGA was expressed and GlcNAc was removed. Using both western blotting and MS additional studies have now found mitochondrial protein GlcNAcylation in other tissues including liver 91, neurons 95, and a variety of cell lines 27. A more specific mechanism of exercise‐induced changes in the body includes the regulation of antioxidants, such as GPX1. This cytosolic and mitochondrial protein was recently identified as a target of *O‐*GlcNAcylation 96. Some of this mitochondrial *O‐*GlcNAcylation regulation in myocytes is associated with changes in the phosphoproteome 92. While other roles of this versatile PTM have been linked to mitochondrial dynamics through *O‐*GlcNAcylation of threonines 585 and 586 of dynamin‐related protein 1 (DRP1) 23, which is required for mitochondrial fission. However, the question as to if the mitochondrial isoform of OGT is sufficient and has the necessary UDP‐GlcNAc substrate available to regulate mitochondrial protein *O‐*GlcNAcylation within the mitochondria has until very recently remained controversial. In a new study, the authors find that rat cardiac mitochondria contain both the 110 kDa mOGT and the 78 kDa soluble isoform of sOGT 97. In addition they show for the first time the presence of OGA in the mitochondria. One of the most exciting findings of this study was the identification of a putative UDP‐GlcNAc transporter (PNC) and evidence for UDP‐GlcNAc uptake into the mitochondria. Together this study provides the first evidence that cardiac mitochondria contain all the necessary proteins and substrate to regulate *O*‐GlcNAcylation 97.

It is now clear that mitochondrial proteins can be *O‐*GlcNAcylated, but a new body of research is suggesting that some of the metabolic changes may result from osmotic stress independent of changes in *O‐*GlcNAc levels 98. Additionally, not all *O‐*GlcNAcylation is maladaptive, as supported by the mortality in the OGT and OGA knockout animals. Other studies have proposed that increased *O‐*GlcNAcylation may play a cardioprotective role, at least in the acute state, context of ischemic injury, or stem cell therapy 99, 100, 101, 102. The role of *O‐*GlcNAcylation appears to be linked to its crosstalk with other PTMs such as phosphorylation in T2D‐associated I/R injury 103. Together these findings suggest that special effort is required to disentangle the highly overlapping PTM milieu. These differences might also be explained by severity and duration of the disease. When comparing cardiac hypertrophy to heart failure, distinct *O‐*GlcNAcylation patterns were found 104, supporting that some modifications may be part of an initially adaptive response. Another explanation for the varying results could simply be the conditions under which samples are collected, as the commonly used anesthesia isoflurane is sufficient to increase protein *O‐*GlcNAcylation 105. We need to further define the mitochondrial *O‐*GlcNAcome if we are to understand the development of diabetic cardiovascular diseases.

### 3.3 Gene regulation and GlcNAc

One of the first roles for *O‐*GlcNAcylation in cellular regulation was by transcriptional control 106, 107. As there are nearly three decades of research in this area please refer to this recent update and the comprehensive reviews cited within 108. In brief, *O‐*GlcNAcylation has been shown to regulate gene expression in most cell types and for most biological pathways including recent progress as it relates to diabetic cardiovascular disease. For example NKX2.5 (NK2 homeobox 5), which is a critical transcription factor in cardiac development, is reduced in the heart following STZ‐induced diabetes and is directly *O‐*GlcNAcylated 29. Myocyte enhancer factor 2 (MEF2), is another critical regulator of cardiac development and regeneration 109. MEF2D is *O‐*GlcNAcylated in cell culture resulting in an inhibition of its transcriptional activity, although MEF2C might not be modified in this cell type 110. These findings suggest additional investigation into these families of myogenic developmental transcription factors is warranted.

Inflammation is a component of cardiac dysfunction and diabetes progression. In the case of T1D, an autoimmune disease, recent evidence found that nuclear factor kappa‐light‐chain‐enhancer of activated B cells (NF‐κB) is *O‐*GlcNAcylated at serine 350 of its c‐Rel subunit 30. These findings pertain directly to the development of T1D through beta cell death. NF‐κB is ubiquitously expressed and this link to hyperglycemia‐induced modifications is an exciting new area to explore. This is particularly true in light of recent evidence showing that inhibition of NF‐κB signaling preserved cardiac function in STZ‐induced diabetes by inhibition of the renin‐angiotensin system and preservation of Ca^2+^ handling 111. Earlier studies found that high glucose‐treated cardiomyocytes had reduced sarcoendoplasmic reticulum Ca^2+^‐ATPase 2a mRNA and protein levels associated with prolonged calcium transients 112. A mechanism that may be regulated by increased *O‐*GlcNAcylation of the transcription factor Sp1 and decreased SERCA2a promoter activity. A third mechanism of *O‐*GlcNAcylation regulating Ca^2+^ handling and transcription is through store‐operated calcium entry (SOCE). This pathway controls Ca^2+^ levels to regulate activity of the transcription factor NFAT, a well‐established regulator of hypertrophic heart disease 113, 114. A critical regulator of SOCE is the protein STIM1 that is GlcNAcylated in cardiomyocytes 115, making this pathway an attractive therapeutic target for treatment of CVD 116. This regulation of calcium handling also occurs through GlcNAcylation of CaMKII in both human and rat diabetic hearts 84, which might contribute to glucose‐mediated induction of arrhythmias seen in diabetic patients.

### 3.4 Histone *O*‐GlcNAcylation

In 2010, *O‐*GlcNAcylation joined the histone code when it was shown that histones 2A, 2B, and 4 were all GlcNAc modified 117. The histone code is a complex interface of histone PTMs (e.g. acetylation, methylation, etc.), DNA modifications (e.g. 5‐methyl cytosine), and microRNAs 118, 119, 120. Each of these factors regulates chromatin to modify gene expression without altering the genetic code. Depending on the location and type of epigenetic mark, these changes can occur acutely in response to a single bout of exercise or be passed on for multiple generations. Just as the mitochondrial proteome can vary between IFM and SSM, the epigenetic signatures of the heart can vary between the left and right ventricles 121. The link between metabolism and epigenetics is becoming increasingly recognized 122, 123, 124, and *O‐*GlcNAcylation as a central nexus of preserving and responding to nutrient changes in the chromatin structure is being explored in cancers 125, neuron development 126, diabetes 124, and heart disease 127.

If excess *O‐*GlcNAcylation is maladaptive in diabetes, one of the more promising aspects to recent findings is that exercise may regulate some of these changes seen in the heart and T1D 128, 129. These studies suggest that lifestyle intervention has the potential to reverse some of the epigenetic marks of past generations. Part of the mechanism by how these changes might be linked to epigenetic regulation came from a study of treadmill running of db/db T2D mice 130. However, in this T2D study exercise further increased total *O‐*GlcNAcylation only in the diabetic mice, but partially attenuated the diabetes‐induced dissociation of OGT and the histone deacetylase HDAC2. The differences between diabetes (e.g. T1D and T2D) and exercise (e.g. swimming and running) in these two studies highlight the need for additional research. Interestingly, exercise capacity in turn might be regulated by *O‐*GlcNAcylation 90. In this study rats selected for low running capacity had insulin resistance, higher total *O‐*GlcNAcylation, and enhanced *O‐*GlcNAcylation of OXPHOS complex I and IV proteins along with VDAC. These studies suggest that *O‐*GlcNAcylation might be a marker of some metabolic programming for mitochondrial function and exercise capacity associated with insulin resistance and cardiac function.

### 3.5 *O*‐GlcNAcylation: Connecting histone and DNA modifications

The interaction between histone PTMs and DNA modifications is an exciting new area of study for GlcNAc biology. In 2009, the ten‐eleven translocation methylcytosine dioxygenase 1 (TET1) was identified for its ability to catalyze the conversion of 5‐methylcytosine to 5‐hydroxymethylcytosine 131. This finding gave a clue as to how DNA might be de‐methylated in epigenetic remodeling. Then in 2013 and 2014, it was found that TET1, TET2, and TET3 all interact with OGT 132, 133, 134, 135, providing an enticing common mechanism linking DNA and histone modifications. In turn all three TETs can be *O‐*GlcNAcylated, but modification of TET3 results in nuclear localization and is enhanced in high glucose conditions 136. Recent advances in this area have found that *O‐*GlcNAcylation of the TET proteins themselves regulates phosphorylation status 137. Specifically, at site resolution it was shown that basal levels of TET phosphorylation are high and these are reduced as *O‐*GlcNAc increased at adjacent amino acids. We have already discussed changes in cardiac *O‐*GlcNAcylation in diabetes and heart failure, but there are also a number of studies now showing that heart failure alters the DNA methylome 138, 139, 140, 141. This link between metabolism, histone *O‐*GlcNAcylation, and DNA methylation is exciting and although these early studies on TET/OGT were not done in the diabetic heart they suggest exciting new avenues of research.

## 4 Other factors

The purpose of this review is to highlight a few of the proteomic mechanisms contributing to heart failure and cardiac dysfunction in diabetes. A number of important contributions have been found and reviewed elsewhere such as contributions from the circadian clock on the cardiac proteome 142, 143, phosphorylation of the cardiac mitochondrial proteome 144, 145, comprehensive modification of the proteasome 146, and the mitochondrial acetylome 147. Some of these PTMs overlap with the *O‐*GlcNAcylation. For example circadian clock proteins are *O*‐GlcNAcylated 148, 149 as well as acetylated 150. This example and all the ones explored above highlights the intricate and important role that PTMs have in controlling the utilization of the very metabolites they derive from in diabetes and heart failure.

### 4.1 Honorable mention: Acetylation

One of the most extensively studied PTMs in heart development and also epigenetic regulation is that of protein acetylation 151. Similar to what has been discussed regarding *O‐*GlcNAcylation, acetylation is tightly regulated by nutrient utilization and has both adaptive and maladaptive implications in regulating cardiac function and therefore must be added to the discussion. The guinea pig cardiac acetylome has been mapped under basal conditions, with mitochondrial proteins accounting for more than half the proteins identified 152. The diabetic mouse liver acetylome was recently published 38 as were those from the livers of fasted and obese mice 153, and it will be exciting to look at these same endpoints in the heart. Tissue‐specific differences in metabolic fuel switching are associated with distinct lysine acetylation patterns on mitochondrial and cytosolic proteins 154. Specifically, looking at the fed to fast transition across six tissues in mice the investigators found two cardiac‐specific increases in acetylation of the mitochondrial proteins, malate dehydrogenase (MDH2) and sarcomeric mitochondrial creatine kinase (S‐MtCK).

The sirtuins (SIRTs) have received particular attention as to their roles in the development of heart failure and aging 155. The activation of SIRTs is linked to intermediary metabolism by the ratio of NAD^+^ to NADH and may therefore act as a sensor of metabolic flux, capacity, and efficiency. Members of the SIRT family are also regulated in diabetic heart disease 156, 157 and respond positively to dietary intervention (e.g. calorie restriction and resveratrol 158, 159), making them attractive therapeutic targets. Recently it was found that SIRT7 deficiency induces mitochondrial dysfunction, particularly in the liver and heart 160. A few of the protein changes resulting from the knockout of this single lysine deacetylase were only seen in the hearts of *Sirt7^−/−^* mice (e.g. reduced ATP5A and UQCRC2).

The acetylation of proteasome complexes reduces their activity and may contribute to the progression of heart failure. These changes are seen in both rodent models following acute I/R injury and in end‐stage heart failure patients at the time of transplant 161. Using high‐resolution LC‐MS/MS, the investigators mapped acetylation on the 20S subunit of the proteasome at sites conserved across rodents and humans. Additional treatment of these samples with HDAC inhibitors (suberoylanilide hydroxamic acid and sodium valproate) resulted in increased acetylation of five of the identified lysines. This change in acetylation status was accompanied by increased proteolytic activity suggesting the exciting possibility of using this approach of HDAC inhibition in heart failure patients. In another similar study it was found that many of the acetylation sites of the 20S subunit are also ubiquitinated 162, supporting additional PTM crosstalk on cardiac proteasomes. Finally, using HDAC inhibitors it is possible to rescue proteolytic function in the failing heart 161.

Cyclophilin D (CypD) is a mitochondrial chaperone protein. When it was genetically removed from the mouse the result was a profound change in cardiac metabolism that is proposed to result from altered acetylation of mitochondrial proteins 25. To map the mitochondrial acetylome, mitochondria isolated from mice lacking CypD were subjected to immunoprecipitation enrichment of acetylated proteins and MS peptide identification. The researchers found that out of the 58 acetylated proteins identified, ten were reduced and 48 were increased in CypD^−/−^ heart mitochondria. This included decreases in acetylation of VDAC proteins and increases in acetylation of multiple OXPHOS proteins supporting an important mechanism of mitochondrial regulation by acetylation. It was mentioned above that *O‐*GlcNAcylation of OXPHOS complex I proteins reduces activity in response to hyperglycemia. It was recently shown that complex I deficiency in turn can increase protein acetylation and cardiac dysfunction 24, suggesting an exciting potential for additional crosstalk between these two pathways in the development of heart failure. Other communication within the cell occurs between the metabolites themselves. Specifically recent evidence has been shown to link cardiac ketone utilization to protein acetylation in heart failure 26, a pathway that should be further explored for *O‐*GlcNAcylation.

## 5 Conclusion

**Figure 2:**
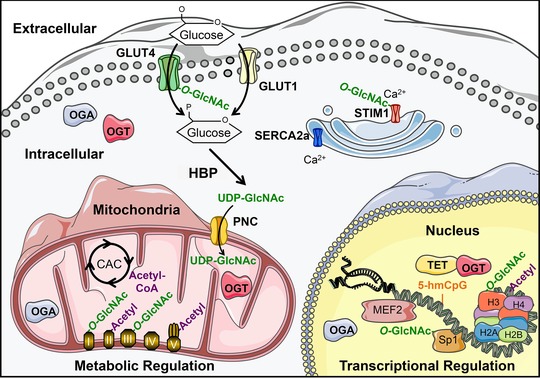
Schematic representation of proteins highlighted for their contributions to molecular regulation in diabetes and heart failure. Regulation of glucose uptake by glucose transporters (GLUT1, GLUT4) can increase flux in the hexosamine biosynthetic pathway (HBP) leading to the generation of UDP‐GlcNAc that can be used for *O‐*GlcNAcylation of proteins by *O‐*GlcNAc transferase (OGT) and *O‐*GlcNAcase (OGA). This includes oxidative phosphorylation complex subunits (I, II, III, IV, and V) as well as proteins involved in calcium handling (STIM1 and SERCA2a). OGA and OGT have now been shown to reside in multiple subcellular organelles including the mitochondria with UDP‐GlcNAc import by the putative transporter PNC. In the nucleus *O‐*GlcNAcylation can occur on both histones (H3, etc.) and transcription factors (e.g. Sp1 and MEF2). Additionally, OGT can interact with TET proteins to regulate DNA methylation via hydroxymethylation (5‐hmCpG). Together this regulation interacts with acetylation fueled by acetyl‐CoA possibly from the citric acid cycle (CAC). Overall, *O‐*GlcNAcylation and acetylation of multiple proteins controls cellular function at both the metabolic and transcriptional levels.

In order to fully understand the molecular changes that occur in obesity and diabetes leading to heart failure, we need to explore the intersection of molecular pathways, particularly the functional aspect of these pathways. This includes a systems biology approach to define how changes in our environment (e.g. diet and exercise) alter metabolite flux in the cell that in turn changes protein function and gene expression in an individual and potentially that individuals offspring. These changes can lead to immediate decline in function (e.g. NDUFA9), alter responses to inflammation and repair (e.g. NF‐κB), regulate calcium handling (e.g. STIM1 and SERCA2a), transcriptional regulation (e.g. Sp1 and MEF2), or reprogram the cell preventing normal cellular function (e.g. epigenetics, *O‐*GlcNAcylation of histones, TET/OGT interaction) (Fig. 2). With the recent identification that the mitochondria has all the machinery necessary for *O‐*GlcNAcylation (Fig. 2), it is clear that this PTM has widespread control of cellular function and may represent one of the primary pathways by which nutrient fluctuations in diabetes alter cardiac function. Harnessing the heart of big data is a new challenge and defining what pieces are included in that analysis is an exponentially increasing task 163. Once we have successfully identified the tissue, developmental, and disease status of each of these PTMs comes the next challenge of integrating the regulation within and between proteins. Some tools are starting to emerge to accomplish this such as PTMcode 164, 165. By continuing the careful and detailed proteomics to define the PTMs and the subsequent molecular biology and biochemistry to define the consequences on function, these and other tools will be one step closer to predicting and aiding in the design of effective therapies for these complex metabolic diseases. The changing proteome offers us one link into how a metabolite such as glucose can alter protein within a short period of time or contribute to epigenetic changes in histone structure and gene expression years down the road. Although not exhaustive this review hopefully leaves us with some insight into recent advances in the measurement and integration of this PTM on the ever‐changing cardiac proteome. The *O‐*GlcNAcome is a critical piece to this clinical puzzle of diabetic cardiomyopathy and altered metabolism is not only leading to an energy‐starved heart but also providing the substrate for post‐translation regulation of the protein machinery itself.

The studies highlighted here have mostly come from animal models and cell culture studies as a result of the difficult task of obtaining human cardiac tissue. This is further confounded by the extremes in epigenetics, environment, duration, treatment strategy, and other racial, age, and sex variables that can confound analysis. It has also been difficult to determine the source of altered *O‐*GlcNAcylation as both excess and deprivation of glucose can alter its levels. Additional research needs to be conducted in clinically relevant patient samples to determine if the same post‐translational mechanisms being defined in model systems can advance biomedical research. While more basic studies need to be conducted to determine the direct role of glucose versus other nutrients in this regulation of protein activity and what if any of these changes persist and alter future cellular function (e.g. epigenetics). The focus on *O*‐GlcNAcylation as a potential therapeutic target is still at its early stages due to conflicting results of both cardioprotective and deleterious effects. Although relatively few clinical trials have begun to look at ways to modulate *O‐*GlcNAcylation, a Phase 2 trial in China (NCT01794884) was conducted to explore if glutamine administration can improve cardiac function following surgery and if this is associated with altered myocardial protein *O‐*GlcNAcylation. However, these results are not yet published. In the meantime, the OGA inhibitor Thiamet G is currently showing promise to alter tau protein hyperphosphorylation in animal models of Alzheimer's disease 166 and may work to help sensitize human leukemia cell lines to chemotherapy 167. There is some early evidence in rodents that this inhibitor may work to define new therapies in heart failure by increasing survival of cardiac stem cells 102. While others have shown that the same increased *O‐*GlcNAcylation might negatively impact troponin T in ischemic heart failure 168. However, until we have a complete proteomic map of the sites of modification and determine both the physiological relevance and the contributions to disease progression we will not be able to fully utilize these interventions.
